# Development of Membrane-Bound GM-CSF and IL-18 as an Effective Tumor Vaccine

**DOI:** 10.1371/journal.pone.0133470

**Published:** 2015-07-17

**Authors:** Chien-Chiao Huang, Kung-Kai Kuo, Ta-Chun Cheng, Chih-Hung Chuang, Chien-Han Kao, Yuan-Chin Hsieh, Kuang-Hung Cheng, Jaw-Yuan Wang, Chiu-Min Cheng, Chien-Shu Chen, Tian-Lu Cheng

**Affiliations:** 1 Department of Biomedical Science and Environmental Biology, Kaohsiung Medical University, Kaohsiung, Taiwan; 2 Department of Surgery, Kaohsiung Medical University Hospital, Kaohsiung, Taiwan; 3 Graduate Institute of Medicine, College of Medicine, Kaohsiung Medical University, Kaohsiung, Taiwan; 4 Graduate Institute of Pharmacognosy, Taipei Medical University, Taipei, Taiwan; 5 Center for Biomarkers and Biotech Drugs, Kaohsiung Medical University, Kaohsiung, Taiwan; 6 Institute of Biomedical Sciences, National Sun Yat-Sen University, Kaohsiung, Taiwan; 7 Department of Surgery, Faculty of Medicine, College of Medicine, Kaohsiung Medical University, Kaohsiung, Taiwan; 8 Department of Aquaculture, National Kaohsiung Marine University, Kaohsiung, Taiwan; 9 School of Pharmacy, China Medical University, Taichung, Taiwan; Duke University Medical Center, UNITED STATES

## Abstract

The development of effective adjuvant is the key factor to boost the immunogenicity of tumor cells as a tumor vaccine. In this study, we expressed membrane-bound granulocyte-macrophage colony-stimulating factor (GM-CSF) and interleukin-18 (IL-18) as adjuvants in tumor cells to stimulate immune response. B7 transmembrane domain fused GM-CSF and IL-18 was successfully expressed in the cell membrane and stimulated mouse splenocyte proliferation. Co-expression of GM-CSF and IL-18 reduced tumorigenesis (*P*<0.05) and enhanced tumor protective efficacy (*P*<0.05) significantly in comparison with GM-CSF alone. These results indicated that the combination of GM-CSF andIL-18 will enhance the immunogenicity of a cell-based anti-tumor vaccine. This membrane-bound approach can be applied to other cytokines for the development of novel vaccine strategies.

## Introduction

A major obstacle in tumor cell vaccine technology is inefficient stimulation of an immune response to induce anti-tumor effects. The co-administration of cytokines is a possible approach for the enhancement of anti-tumor immunity. Various cytokines have been tested for their host immune stimulation activity for cancer treatment, such as IL-2, GM-CSF, and INF-α[1]. Among these, GM-CSF has been widely studied and has shown promising anti-tumor results in many tumor models, such as melanoma cells[2], bladder cancer cells[3], murine leukemia[4], etc. GVAX (Cell Genesys) is a tumor vaccine comprised of genetically modified tumor cells engineered to secrete GM-CSF. It has been studied in a number of cancer types in preclinical and clinical trials[5], and demonstrated promising results in both phase I and II clinical trials of pancreatic and prostate cancer patients [6–8]. However, a phase III trial of GVAX was prematurely terminated because of the inability to meet the survival advantages[9–10].Thus, to enhance the stimulatory effects of GM-CSF might be important for further vaccine development.

The combination of GM-CSF and a second cytokine might be an effective approach to improve the anti-tumor response. GM-CSF is regarded to be ideal adjuvant owing to its potent activation of dendritic cells (DC) and myeloid progenitor maturation. GM-CSF secreting tumor vaccines have been reported to induce massive accumulation of DCs at the inoculated site and in turn to activate tumor specific T cells to induce an anti-tumor response[11–15]. A second cytokine aimed at stimulating lymphoid cells might be important to further augment the immune response. IL-18 has been reported to effectively enhance Th1 immunity and tumor protection in murine models[16–17]. In addition, IL-18was also reported to enhance the proliferation and cytotoxic activity of both T cells and NK cells[18–22]. Thus, IL-18 may be a good candidate to enhance the effects of GM-CSF.

**Fig 1:**
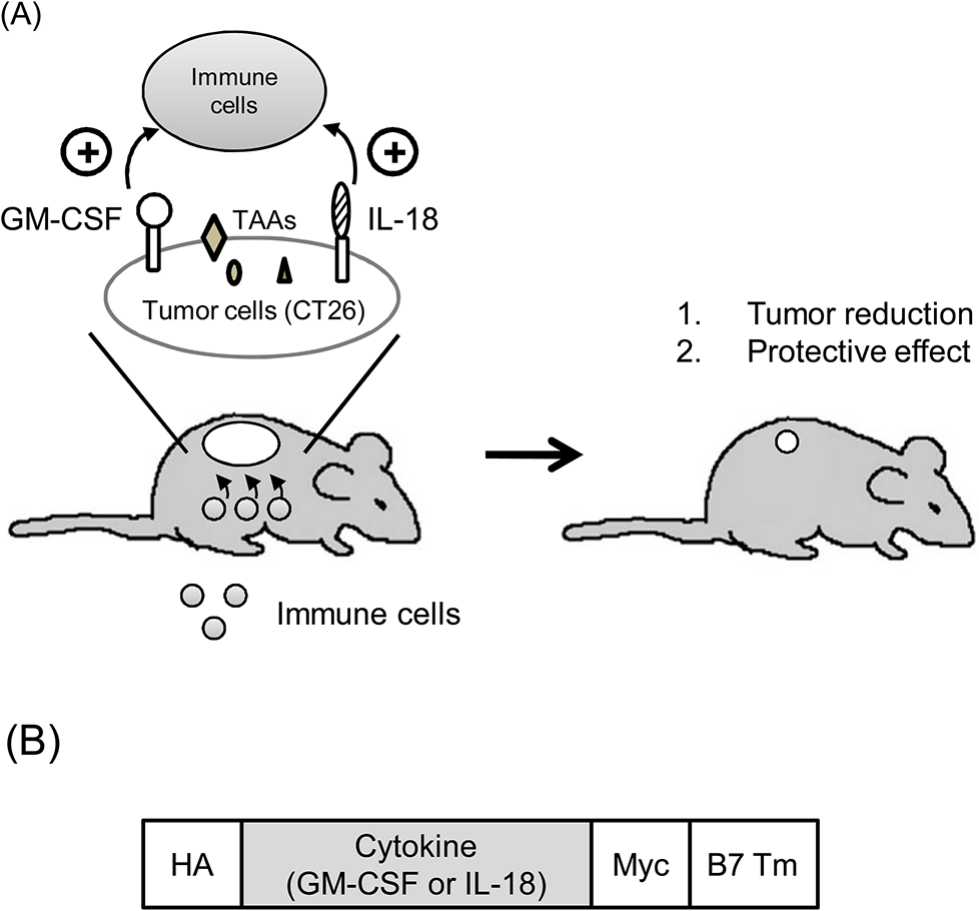
Schematic representation of a membrane-bound strategy for immune stimulation. (A) The establishment of genetically modified CT26 cells to express membrane-bound GM-CSF and IL-18. Co-expression of GM-CSF and IL-18 on CT26 cells induced massive accumulation of immune cells at the inoculated site and stimulated in a locally specific manner. Tumor reduction and protective effects was then assessed for evaluation of the immune stimulatory effects. (B) The membrane-bound GM-CSF and IL-18 were composed of a HA epitope tag, the cytokine gene, a Myc tag, and the B7 transmembrane domains (B7 Tm). TAA, tumor associated antigen.

In this study, we co-expressed GM-CSF and IL-18 in colon carcinoma cells (CT26) and examined the anti-tumor effects compared with GM-CSF alone (Fig 1A). We first generated the membrane-bound GM-CSF and IL-18 by fusion with the B7 transmembrane domain and the protein expression level was determined by flow cytometry. The bioactivity of membrane-bound GM-CSF and IL-18 was confirmed by the stimulation of mouse spleen cell proliferation. The tumor inhibition and tumor protection effects of GM-CSF were then investigated with or without IL-18. The results suggested that IL-18 might enhance the therapeutic potency of GM-CSF. In addition, the flexibility of this membrane-bound platform may facilitate the development of novel vaccine strategies.

## Materials and Methods

### Cells and animals

The mouse colon carcinoma cell line CT26 and the retroviral packaging cell line GP2-293 were purchased from American Type Culture Collection (ATCC). The cells were cultured in Dulbecco’s modified Eagle’s medium (DMEM; Sigma-Aldrich) supplemented with 10% heat-inactivated bovine calf serum, 100 units/mL penicillin, and 100 m g/mL streptomycin (Sigma-Aldrich) at 37°C in a humidified atmosphere containing5% CO_2_. Balb/cByJNarl mice (6 to 12 weeks old) were obtained from the National Laboratory Animal Center, Taipei, Taiwan. In the end of experiments, mice were sacrificed by CO_2_ asphyxiation. All animal experiments were carried out in accordance with institutional guidelines and approved by the Animal Care and Use Committee of the Kaohsiung Medical University, Kaohsiung, Taiwan.

### Construction and establishment of membrane-bound cytokine IL-18 and GM-CSF expressing CT26 cells

The precise cDNA sequence of murine IL-18 or GM-CSF followed by that of the B7 transmembrane domain was subcloned into the retroviral vector pLNCX (BD Biosciences, San Diego, USA) using a standard procedure (Fig 1B). Recombinant retroviral particles were packaged by co-transfection of pVSVG with pLNCX constructs into GP2-293 cells (Clontech, USA). After 48 hours, the harvested culture medium was filtered through a 0.22-μm syringe filter, followed by mixing with polybrene to 8 μg/ml. It was then added to CT26 colon carcinoma cells for virus infection. The stable CT26 cells were selected by G418 and were sorted by FACScaliber flow cytometer to establish CT26/IL-18, CT26/GM-CSF and CT26/GM-CSF/IL-18 clones.

### Determination of expression of membrane-bound IL-18 and GM-CSF

The transduced CT26 cells (CT26/IL-18, CT26/GM-CSF and CT26/GM-CSF/IL-18) were resuspended in a polystyrene tube (Falcon, USA) at concentration of 1×10^6^ cells/ml in phosphate buffered saline (PBS). Cells were stained with 5 μg/ml mouse anti-HA antibody for 30 min, followed by 5μg/ml FITC-conjugated goat anti-mouse IgG for 30 min. After extensive washing, the florescence of the cells was measured and sorted by a FACScaliber flow cytometer. For double staining, 1×10^6^ CT26/GM-CSF/IL-18 were first incubated with goat anti-mouse IL-18 IgG (Santa Cruz) and rat anti-mouse GM-CSF IgG (BioLegend) for 30 min. Then Donkey anti-goat IgG (L+H)-FITC (Jackson) and goat anti-rat IgG-PE (Jackson) were added sequentially for another two rounds of 30 min incubation. Three times of washes is needed between each incubation steps to remove the unbounded antibodies. After extensive washing, the florescence intensity was measured.

### Determination of growth curves of membrane-bound cytokine IL-18 and GM-CSF expressing CT26 cells

For in vitro proliferation assay, transduced CT26 were seeded into 12-well cell plates at density of 5×10^4^ cells in 1 ml culture media. The CT26 cells were harvested by mixture of 100 μl of 0.05% trypsin contained PBS, and then added with 900 μl 10% BCS contained DMEM. The number of viable cells of each group was counted with the hemocytometer every twenty-four hours. Independent experiments were repeated three times.

### Splenocyte proliferation assay for the bioactivity of membrane-bound IL-18 and/or GM-CSF

CT26 cells were suspended in PBS at a concentration of 1×10^6^ cells/ml and continuously freeze-thawed 5 times in liquid nitrogen and a water bath at 37°C. Balb/C mice were injected i.p. with 50μl of prepared CT26, followed by a second injection 7 days after the first injection. 10 days after the first immunization, mice were sacrificed by CO_2_ before the spleens were harvested. Spleens were mashed and filtered through a cell strainer, followed by treatment with ACK lysis buffer for RBC removal. After extensive washing, 1×10^5^ splenocytes were seeded in 96 well plates containing 200 μl medium per well. After co-incubation with 1×10^4^ irradiated transduced CT26 cells, ATPlite luminescence assay (PerkinElmer) was performed at the indicated time point according to manufacturer’s instructions.

### Determination of the tumorigenicity of IL-18 and/or GM-CSF-expressing CT26 cells

Transduced CT26 cells were washed 5 times in PBS before injection. The CT26 cells were suspended at the indicated concentrations and injected with 0.1 ml PBS. A group of BALB/c (n = 5) mice were injected s.c. in the right hind leg with 1×10^6^ cells of either transduced or mock-transduced CT26 cells (CT26/IL-18, CT26/GM-CSF, and CT26/GM-CSF/IL-18). Tumor volume (length × width × height × 0.5) was estimated from tumor-bearing mice every 3 or 4 days after injection. Mice were sacrificed by CO2 asphyxiation when tumors reached a maximal size of 2,000 mm^3^.

### Determination of the protective effects of IL-18 and/or GM-CSF expressing CT26 cells

After suspension, transduced CT26 cells were washed 5 times in PBS before injection. The CT26 cells were suspended at the appropriate concentration (1×10^7^ cells/ml) in PBS, and then received 30 Gy (1 Gy = 100 rads) radiation before vaccination. A group of BALB/c mice (n = 5) were injected s.c. in the right hind leg with 1×10^6^ radiated cells of transduced or mock-transduced CT26 in 100 μl PBS. Ten days after tumor cell vaccination, mice were challenged by s.c. injection with 5×10^5^ mock-transduced CT26 cells in the left hind leg. Tumor volume (length × width × height × 0.5) and tumor-free survival rate was estimated every 3 or 4 days after challenge. Tumor-free survival rate of each group was calculated as the cumulative number of tumor–free mice per total number of mice. Of note, all the mice survived until the end of the assay. Independent experiments were repeated three times. Mice were sacrificed by CO2 asphyxiation when tumors reached a maximal size of 2,000 mm^3^.

### Statistical Analysis

Statistical analysis was performed by using Graphpad Prism V5 software. All data was analyzed for significance by using One-way ANOVA followed by Student’s Newman-Keuls test. Statistical significance of differences was considered when P value was <0.05.

## Results

### Establishment of IL-18 and/or GM-CSF stably expressing CT26 cells

**Fig 2:**
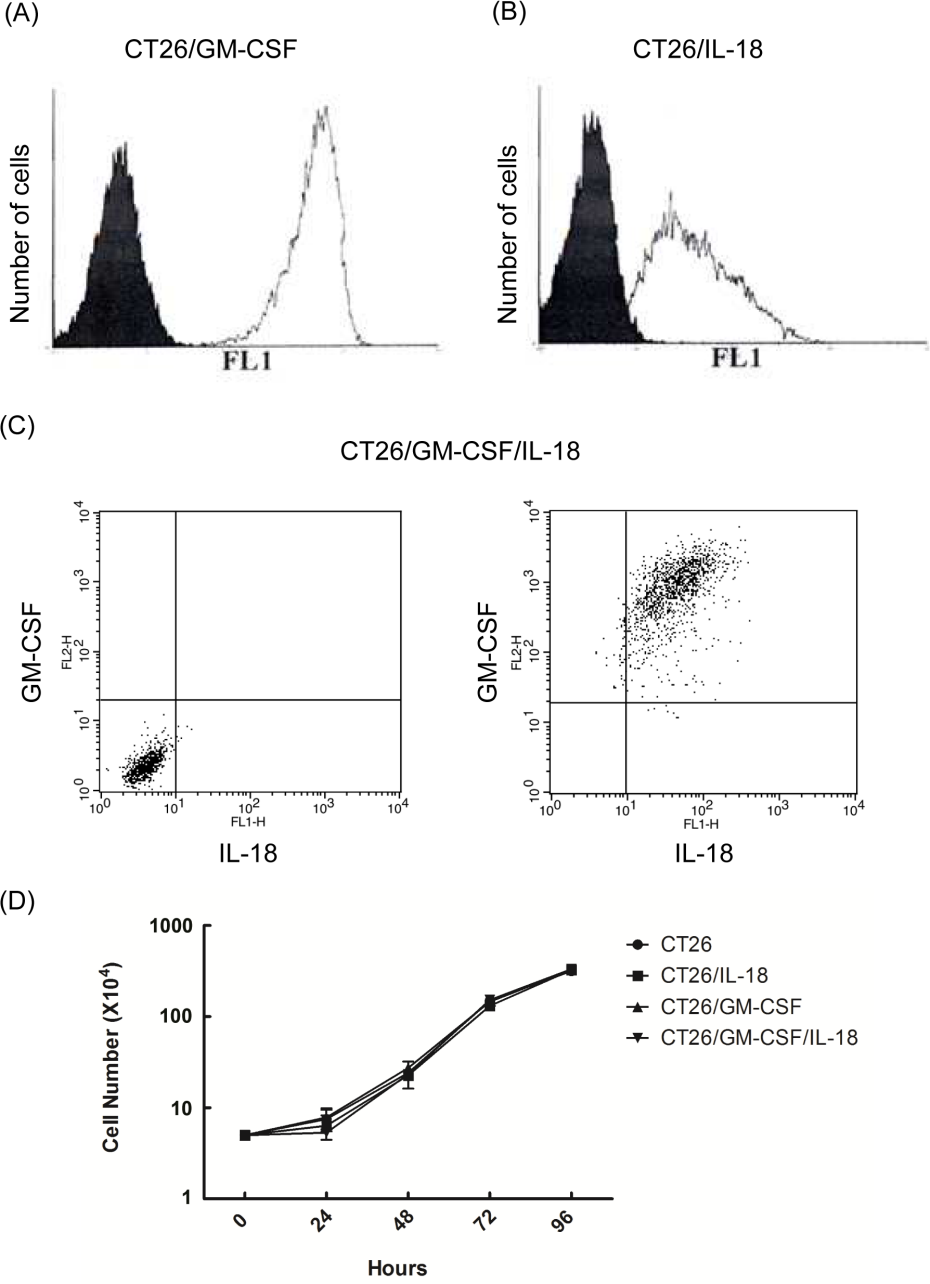
Protein expression of membrane-bound GM-CSF and IL-18. CT26 cells stably expressing GM-CSF (A) or IL-18 (B) were stained with mouse anti-HA antibody, followed by FITC-conjugated goat anti-mouse IgG for 30 min. Mock-transduced CT26 cells were used as a negative control (black). (C) CT26/GM-CSF/IL-18 was doubly stained by anti-GM-CSF and anti-IL-18 primary antibodies, followed by FITC and PE-conjugated secondary antibodies (Experimental details were described in Materials and Methods). The left panel is negative control, in which CT26/GM-CSF/IL-18 was stained by secondary antibody alone. The right panel is the result of double staining, in which CT26/GM-CSF/IL-18 cells was stained with first and secondary antibodies. Fluorescence intensity of membrane-bound cytokines was analyzed by flow cytometry. (D) Mock-transduced CT26, CT26/GM-CSF, CT26/IL-18, and CT26/GM-CSF/IL-18 cells were seeded into 12-well plates at the density of 5×10^4^ cells per well. The number of viable cells of was counted with the hemocytometer every twenty-four hours. Independent experiments were repeated three times.

To assess the effect of adding IL-18 to GM-CSF, membrane-bound GM-CSF and IL-18 expressing CT26 cells were established. The murine GM-CSF and IL-18 genes, flanked by HA and Myc tag, were subcloned into the upstream of the B7 transmembrane domain (B7 Tm) in the retroviral vector pLNCX (Fig 1B). pLNCX/GM-CSF, pLNCX/IL-18, and pLNCX/GM-CSF/IL-18 plasmids were transfected into packaging cells for virus production. The virus was then harvested and infected into CT26 cells for surface expression of cytokines. After the selection of G418, stable cytokine expressing cells, CT26/GM-CSF,CT26/IL-18, and CT26/GM-CSF/IL-18, were established. The expression of surface GM-CSF and IL-18 was examined by flow cytometry. CT26/GM-CSF,CT26/IL-18, and CT26/GM-CSF/IL-18 cells revealed strong fluorescence intensity compared with the negative control, and demonstrated membrane-bound GM-CSF and IL-18 was successfully expressed on the CT26 cells (Fig 2A–2C). To determine whether the expression of membrane-bound GM-CSF and/or IL-18 impairs cell growth of CT26 cells, in vitro proliferation assay was conducted. As shown in Fig 2D and S1 Table, the growth curves of these transduced CT26 cell lines are quite similar. Very low amount of Trypan blue positive cells were observed (average 1–10 cells each observation), which indicated no abnormal cell death was occurred. These results revealed that membrane-bound GM-CSF and/or IL-18 do not impair the CT26 cell proliferation/survival in vitro.

### Confirmation of the bioactivity of membrane-bound GM-CSF and IL-18 by splenocyte proliferation assay

**Fig 3:**
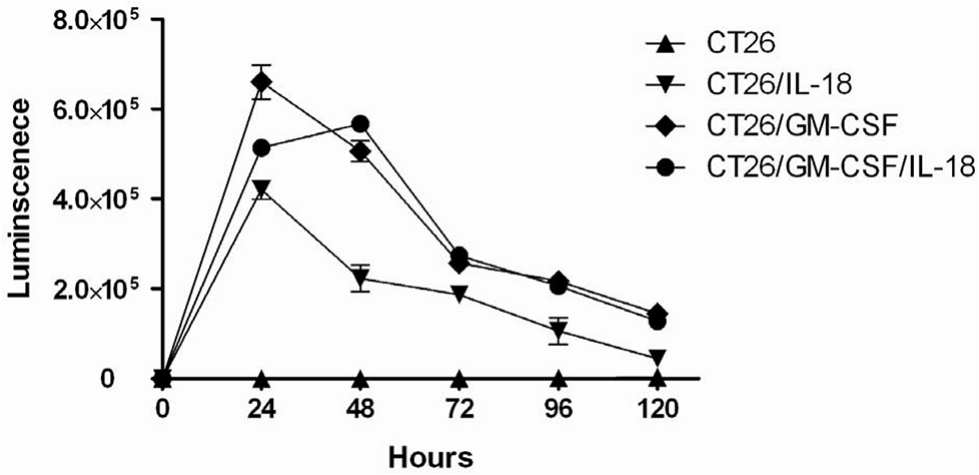
Splenocyte proliferation assay for the bioactivity of membrane-bound IL-18 and/or GM-CSF. Splenocytes were harvested from mock-transduced CT26 immunized Balb/C mice, and were cultured with CT26/IL-18, CT26/GM-CSF, and CT26/GM-CSF/IL-18 cells for the indicated times. ATPlite luminescence assay was performed to measure the splenocytes proliferation.

Next, to determine the bioactivity of these membrane-bound cytokines, we assessed the effects of CT26/IL-18, CT26/GM-CSF, and CT26/GM-CSF/IL-18 on mouse splenocytes proliferation. Splenocytes were isolated from mice immunized with the repeated freeze-and-thawed CT26 cells. After co-incubation with CT26/GM-CSF, CT26/IL18, and CT26/GM-CSF/IL-18, cell number was calculated at the indicated time points (Fig 3 and S2 Table). Compared with the negative control, significant proliferative response was observed in all three membrane cytokine expressing CT26 cells. The number of splenocytes stimulated by CT26/GM-CSF and CT26/GM-CSF/IL-18 reached to 5–7×10^5^ in 24 hours and was rapidly reduced after 48 hours. Similarly, splenocytes incubated with CT26/IL-18 increased to 4×10^4^ cells in 24 hours, but quickly decreased after 24 hours. Compared with GM-CSF alone, no enhanced stimulation of cell proliferation was observed with co-expression of IL-18 and GM-CSF. This data confirmed the bioactivity of membrane-bound GM-CSF, IL-18, and GM-CSF and IL-18 co-expression.

### Tumorigenesis of CT26/GM-CSF, CT26/IL-18, and CT26/GM-CSF/IL-18cells

**Fig 4:**
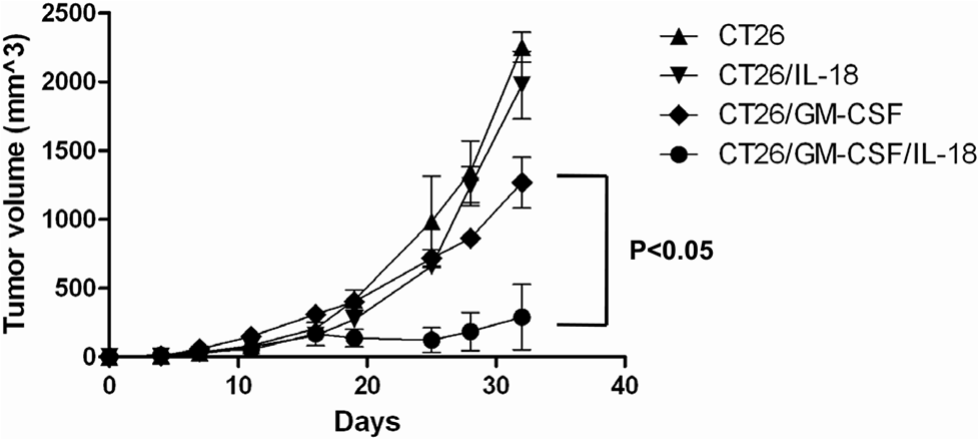
Tumorigenicity of membrane-bound GM-CSF, IL-18, and GM-CSF/IL-18 expressing CT26 cells. Groups of (n = 5) BALB/c mice were injected with 1 ×10^6^ transduced tumor cells s.c. in the right hind leg. Tumor volume (length × width × height × 0.5) was collected from tumor-bearing mice every 3 or 4 days after injection. Independent experiments were repeated three times.

To investigate the effects of IL-18 on GM-CSF on tumorigenesis, tumor growth of membrane-bound cytokine expressing CT26 cells was examined in a mouse model. CT26/GM-CSF, CT26/IL18, and CT26/GM-CSF/IL-18 cells (1 ×10^6^ of each) were inoculated subcutaneously into Balb/C mice, and tumor volume was monitored from tumor-bearing mice every 3 or 4 days. As shown in Fig 4 and S3 Table, membrane-bound GM-CSF reduced the growth of CT26 tumor cells in comparison with control cells. Co-expression of membrane-bound IL-18 and GM-CSF significantly enhanced the tumor inhibitory effects of GM-CSF (*P*<0.05). Membrane-bound IL-18 alone had no effect on tumor formation, but an enhanced anti-tumor response was observed when it was combined with GM-CSF.

### Tumor protective effects of CT26/GM-CSF, CT26/IL-18, and CT26/GM-CSF/IL-18 in vivo

**Fig 5:**
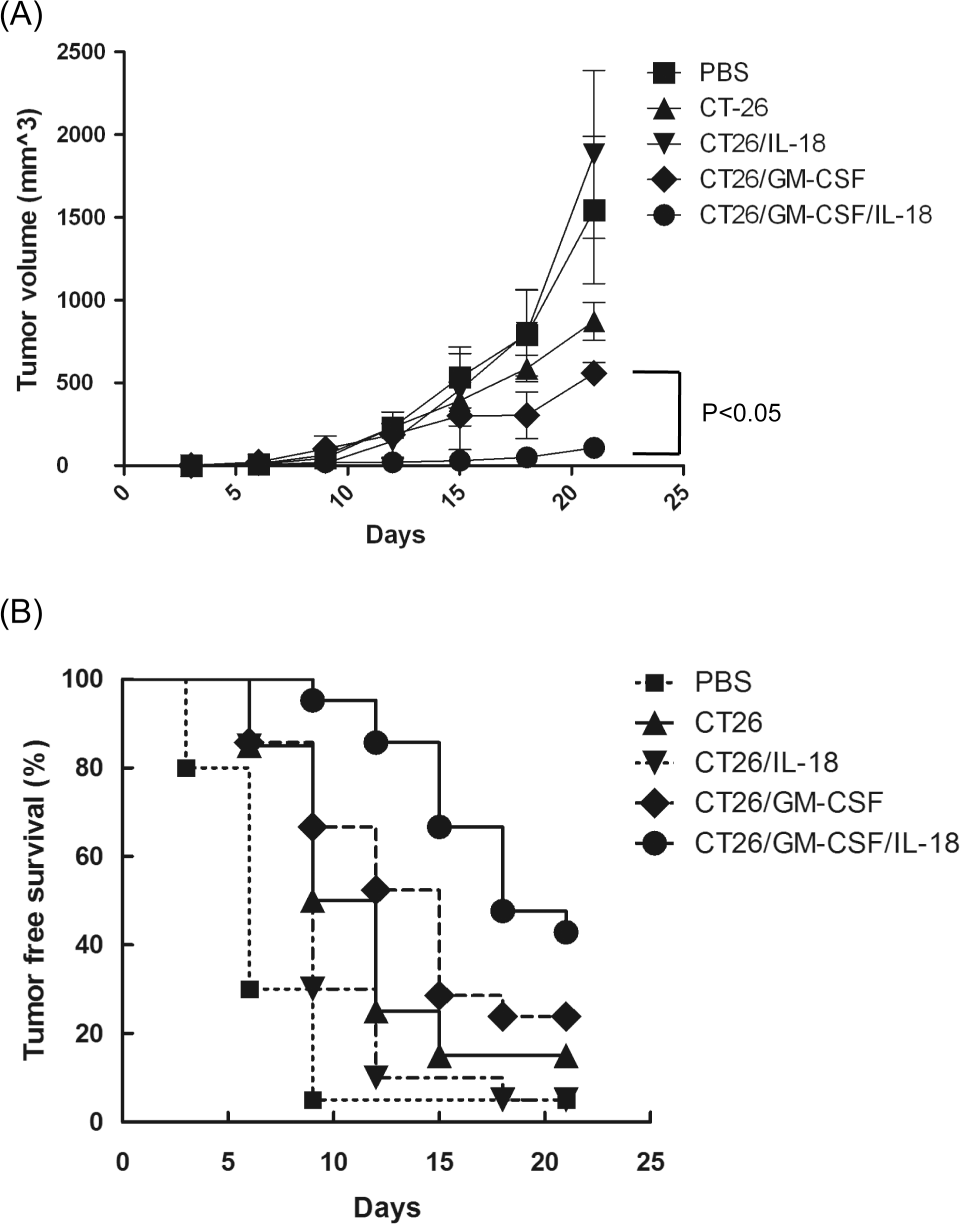
Protective effects of membrane-bound GM-CSF and IL-18, and GM-CSF/IL-18 expressing CT26 cells. (A) Groups of BALB/c mice (n = 5) were injected s.c. in the right hind leg with 1 × 10^6^ transduced tumor cells. Ten days after tumor cell implantation, mice were challenged by s.c. injection with 5×10^5^ mock-transduced CT26 cells in the left hind leg. Tumor volume (length × width × height × 0.5) was estimated every 3 or 4 days after challenge. Independent experiments were repeated three times. (B) Tumor free survival rate was estimated simultaneously (n = 20). The tumor free survival rate of respective group is about 40% (CT26/GM-CSF/IL-18), 25% (CT26/GM-CSF), 15% (CT26), and less than 5% (CT26/IL-18 and PBS control)

To assess the effects of membrane-bound IL-18 and GM-CSF on systemic tumor protection, mice were re-challenged with mock-transduced CT26 after immunization with irradiated cytokine expressing CT26 cells. As shown in Fig 5A and S4 Table, vaccination of mock-transduced CT26 cells provided protective effects when re-challenged with parental tumor cells in the other leg ten days after vaccination. An enhanced effect was observed when vaccination with CT26/GM-CSF compared to mock-transduced CT26. In contrast, CT26/IL-18 revealed no tumor protection effects and showed a similar degree of tumor inhibition to PBS control. Importantly, CT26/GM-CSF/IL-18 reduced tumor growth significantly in comparison with CT26/GM-CSF (*P*<0.05). These results suggested that the anti-tumor immunity elicited by CT26/GM-CSF can act systemically and inhibit tumors at a distant site, and the protection effects of GM-CSF were further amplified when it was used in combination of IL-18. In the end of tumor protective assay, the tumor free survival rate of respective group also showed similar results, which is about 40% (CT26/GM-CSF/IL-18), 25% (CT26/GM-CSF), 15% (CT26), and less than 5% (CT26/IL-18 and PBS control) (Fig 5B and S5 Table). Taken together, these results indicated that IL-18 might be a good candidate as an adjuvant to enhance the tumor protective effects of GM-CSF.

## Discussion

In this study we demonstrated that membrane-bound IL-18 may increase anti-tumor effects used in combination with GM-CSF. Co-expression of IL-18 significantly reduced tumor growth (*P*<0.05) and improved the tumor protective effects (*P*<0.05) when compared with GM-CSF alone. Based on these results, the co-expression of GM-CSF and IL-18 may revive the possibility that GM-CSF can be used clinically. Additionally, our membrane-bound approach had higher flexibility to be easily applied to various secreted cytokines and cancer models. Thus, we believe this strategy may be useful in the development of novel vaccines.

Compared with soluble cytokines, the use of membrane-bound cytokines as an adjuvant has various potential advantages. The expression of membrane-bound cytokines allows for local activation of immune response at the vaccination site rather than systemic activation, which might potentially be harmful to patients. In contrast, strategies that either combine soluble proteins as an adjuvant or genetically modify tumor cells for cytokine secretion face potential problems caused by non-specific binding[23]. Several side-effects caused by systemic administration have been reported. Faries and colleagues showed the adverse outcome when GM-CSF was administered as an allogeneic whole-cell melanoma vaccine. Systemic recruitment of eosinophils and basophils was observed and accompanied by a trend toward worse survival[24]. In a study by Hazenberg and colleagues, soluble GM-CSF was tested to correct granulocytopenia in patients with Felty’s syndrome. However, an unpredictable flare-up of arthritis of all joints and highly elevated IL-6 circulation was observed [25]. Similarly, Campbell and colleagues reported that GM-CSF exacerbated collagen-induced arthritis in a mouse model[26]. Thus, a strategy to stimulate immune response locally and specifically at the vaccination site may be important for vaccine safety. A previous study evaluated the anti-tumor effects of soluble GM-CSF and IL-18 expressing Lewis lung cancer tumor cells LL/2[27], the non-specific delivery problem might need to be solved. In this study, the co-expression of membrane-bound GM-CSF and IL-18 enhanced anti-tumor immunity without the potential risks of systemic toxicity of soluble cytokines. Thus, the use of membrane-bound cytokines specifically acting on immune cells that infiltrate at vaccination site may be beneficial for vaccine safety.

In addition, the local activation of anti-tumor response promotes systemic immune protection specific to tumor cells [28]. Locally accumulated stimulatory cytokines have also been reported to alter the tumor microenvironment, resulting in tumor reduction, and induction of prolonged protective immunity [29–30]. Soluble cytokines are hard to accumulate using conventional strategies. Using the membrane-bound strategy, cytokines were enriched on the cell membrane and were longer-lasting, which may also enhance the immune response for a vaccine. Moreover, direct targeting of tumor-associated antigens (TAAs) to antigen presenting cells (APC) is more likely to occur with enriched membrane-bound GM-CSF on tumor cells and their receptors on DCs. Taken together, we believe that the expression of cytokines in a locally specific and concentrated manner may be one key to the enhancement of adjuvant effects. Pan and colleagues expressed membrane-bound IL-12 with the use of the B7-1 transmembrane and cytoplasmic domains. They demonstrated that membrane-bound IL-12 has the advantage of minimizing circulatingIL-12 without compromising its antitumor efficacy [31]. Bozeman and colleagues used GPI to anchor IL-2 and IL-12 on the membrane of murine mammary tumor cells as a tumor vaccine. Their results showed that these modified tumor cells effectively induced protective immunity, inhibited the growth of distant tumors, and overcame the immune suppressive microenvironment [29]. Thus, this membrane-bound strategy may improve the therapeutic potency and reduce the nonspecific toxicities of traditional vaccine adjuvants.

The selection of anchor protein for surface expression is important for protein stability. Several advantages of the B7 transmembrane domain to anchor the secreted protein have been reported. Liao and colleagues demonstrated that B7-containing AFP chimeric protein displayed a longer half-life (about 12.2 h) compared with other targeting domains including DAF (3.8 h), ASGPR (2.4h), or PDGFR (1.6 h), and indicated that the B7 fused protein is stable and correctly folded. They also showed that AFP-B7 fusion protein was rapidly sorted to the cell surface and did not undergo endocytosis[32].Our results also demonstrated that the B7 transmembrane domain fused cytokines were highly expressed on the cell membrane and remained bioactive. Thus, the B7 transmembrane domain may be a good target for the membrane-bound strategy. In addition, if needed, the protein expression level and ratio can be further adjusted to achieve the best efficacy by cell sorting techniques. Using this platform, membrane-bound cytokines can be changed rapidly for various vaccine strategies. Also, this strategy could be applied to other tumor models easily because of the stability and high expression rate of the B7 fused protein.

The diversity of TAAs is a major challenge in vaccine development. Because of the lack of knowledge of TAAs in most human cancers, the use of tumor cells as a broad antigen source is currently considered to be the most effective means of presenting antigens[33]. Whole-cell tumor vaccines have been investigated for more than 20 years in both animal models and in human clinical trials, and various advantages have been reported[34]. First, unknown or mutated tumor antigens can be targeted by tumor vaccines. Second, whole tumor cell vaccines have been reported to target to both the innate and adaptive immune system for a better immune response [5, 35–36]. In contrast, protein-based vaccines have been reported to induce a stronger response on the generation of CD4^+^ T cells, but not CD8^+^ T cells, which is not beneficial for tumor clearance[37]. The weak immunogenicity of single peptides and tumor immune evasion through antigen point mutation has also been mentioned previously[38]. Thus, we chose whole tumor cells as the major source of TAAs, to provide a higher efficacy and lower cost method to re-activate the immunogenicity of tumor cells. However, one limitation of whole tumor vaccine is the possibility of eliciting autoimmune or allergic responses in both tumor-associated and self-antigens exposed to immune cells at the same time.

In this study, we successfully enhanced the effects of GM-CSF on CT26 tumor cells by co-expression with membrane-bound IL-18. Recently, the enhanced anti-tumor effect was also reported on Lewis lung cancer cells LL/2 modified to co-express soluble GM-CSF and IL-18 as a tumor vaccine[27]. The mechanism was reported mainly to be dependent on CD4^+^ and CD8^+^ T cells. In our study, the anti-tumor mechanism of membrane-bound GM-CSF and IL-18 might be similar. Of note, our membrane-bound strategy revealed several advantages: (1) Membrane-bound approach minimized circulating cytokines without compromising its efficacy and lowered the risk of potential side-effects caused by non-specific binding of cytokines; (2) B7 fused protein is stable, expressed at high levels in the membrane, and less internalized. It will, therefore, provide longer-lasting and stronger stimulation to effecter cells; (3) Protein expression level and ratio can be easily adjusted to provide optimal anti-tumor effects; (4) Both cytokines and tumor models are selectable and conveniently exchangeable. With such flexibility and stability, we believe that this platform is valuable for novel vaccine development and the revival of the GM-CSF vaccine in clinical trials.

## Supporting Information

S1 Table Determination of growth curves of membrane-bound cytokine IL-18 and GM-CSF expressing CT26 cells. Mock-transduced CT26, CT26/GM-CSF, CT26/IL-18, and CT26/GM-CSF/IL-18 cells were seeded into 12-well plates at the density of 5×10^4^ cells per well. The number of viable cells (Trypan Blue negative) was counted with the hemocytometer every twenty-four hours. Independent experiments were repeated three times.
(XLSX)

S2 Table Splenocyte proliferation assay for the bioactivity of membrane-bound IL-18 and/or GM-CSF. Splenocytes were harvested from mock-transduced CT26 immunized Balb/C mice. 1×10^5^ splenocytes were cultured with 1×10^4^ CT26/IL-18, CT26/GM-CSF, and CT26/GM-CSF/IL-18 cells for the indicated times. ATPlite luminescence assay was performed to measure the splenocytes proliferation
(XLSX)

S3 Table Tumorigenicity of membrane-bound GM-CSF, IL-18, and GM-CSF/IL-18 expressing CT26 cells. Groups of (n = 5) BALB/c mice were injected with 1 ×10^6^ transduced tumor cells s.c. in the right hind leg. Tumor volume (length × width × height × 0.5) was collected from tumor-bearing mice every 3 or 4 days after injection.
(XLSX)

S4 Table Protective effects of membrane-bound GM-CSF and IL-18, and GM-CSF/IL-18 expressing CT26 cells. Groups of BALB/c mice (n = 5) were injected s.c. in the right hind leg with 1 × 10^6^ transduced tumor cells. Ten days after tumor cell implantation, mice were challenged by s.c. injection with 5×10^5^ mock-transduced CT26 cells in the left hind leg. Tumor volume (length × width × height × 0.5) was estimated every 3 or 4 days after challenge.
(XLSX)

S5 Table Tumor free survival rate of tumor protective assay. Groups of BALB/c mice (n = 20) were injected s.c. in the right hind leg with 1 × 10^6^ transduced tumor cells. Ten days after tumor cell implantation, mice were challenged by s.c. injection with 5×10^5^ mock-transduced CT26 cells in the left hind leg. Tumor volume (length × width × height × 0.5) was estimated every 3 or 4 days after challenge.
(XLSX)
